# Upper Extremity Dedifferentiated Liposarcoma With an Osseus Component: An Uncommon Pathology

**DOI:** 10.7759/cureus.73735

**Published:** 2024-11-15

**Authors:** Emmanuel Ihionkhan, Vincent Marcucci, Victoria Grille, Peter Alexander, John Gibbs

**Affiliations:** 1 Neurosurgery, Hackensack University Medical Center, Hackensack, USA; 2 Surgery, Jersey Shore University Medical Center, Neptune, USA; 3 Surgery, St. George's University School of Medicine, St. George's, GRD

**Keywords:** basic oncology, histochemisty, orthopedic, sarcoma, surgery

## Abstract

Liposarcomas are the most common soft tissue sarcoma primarily originating in deep soft tissues and the retroperitoneum. Sarcoma classification includes atypical lipomatous tumor/well-differentiated liposarcoma (WDL) and dedifferentiated liposarcoma (DDL), myxoid liposarcoma, and pleomorphic liposarcoma. DDL is most prevalent in the retroperitoneum and often has two distinct components, a well-differentiated lipomatous component and a dedifferentiated nonlipomatous component that could be morphologically similar to malignant fibrous histiocytoma (MFH) or fibrosarcoma. DDLs can undergo heterologous differentiation into multiple cancer types including rhabdomyosarcoma, chondrosarcoma, melanoma, and leiomyosarcoma. Rarely, DDLs can undergo metaplastic bone formation. We report a peculiar case of a DDL with an osseous component.

## Introduction

Liposarcomas are the most common soft tissue sarcomas, accounting for approximately 20% of malignant mesenchymal neoplasms [1]. They are classified into three major categories: atypical lipomatous tumors/well-differentiated liposarcoma (WDL) and dedifferentiated liposarcoma (DDL), myxoid liposarcoma, and pleomorphic liposarcoma [2]. The largest subgroups of liposarcomas are WDLs and DDLs, accounting for 40-45% of all liposarcomas [1]. 

DDL is a high-grade non-lipogenic sarcoma that most commonly occurs in the setting of a preexisting WDL and, less commonly, a de novo tumor [3]. DDLs primarily occur in adults over 50 years of age and have a slight male preponderance [3]. The predominant histological appearance of a DDL is characterized by pleomorphic malignant fibrous histiocytoma-like features, with less common occurrences of sarcomatous histology, including instances of ossification [4]. The management of DDL requires surgical resection with a 41-52% local recurrence rate and a five-year disease-specific survival of 44% [5]. There are few reports of an osseous element of DDL in the upper extremity. Here, we present the case of a 30-year-old patient who underwent a local DDL resection with an osseous component in the right upper extremity.

## Case presentation

**Figure 1:**
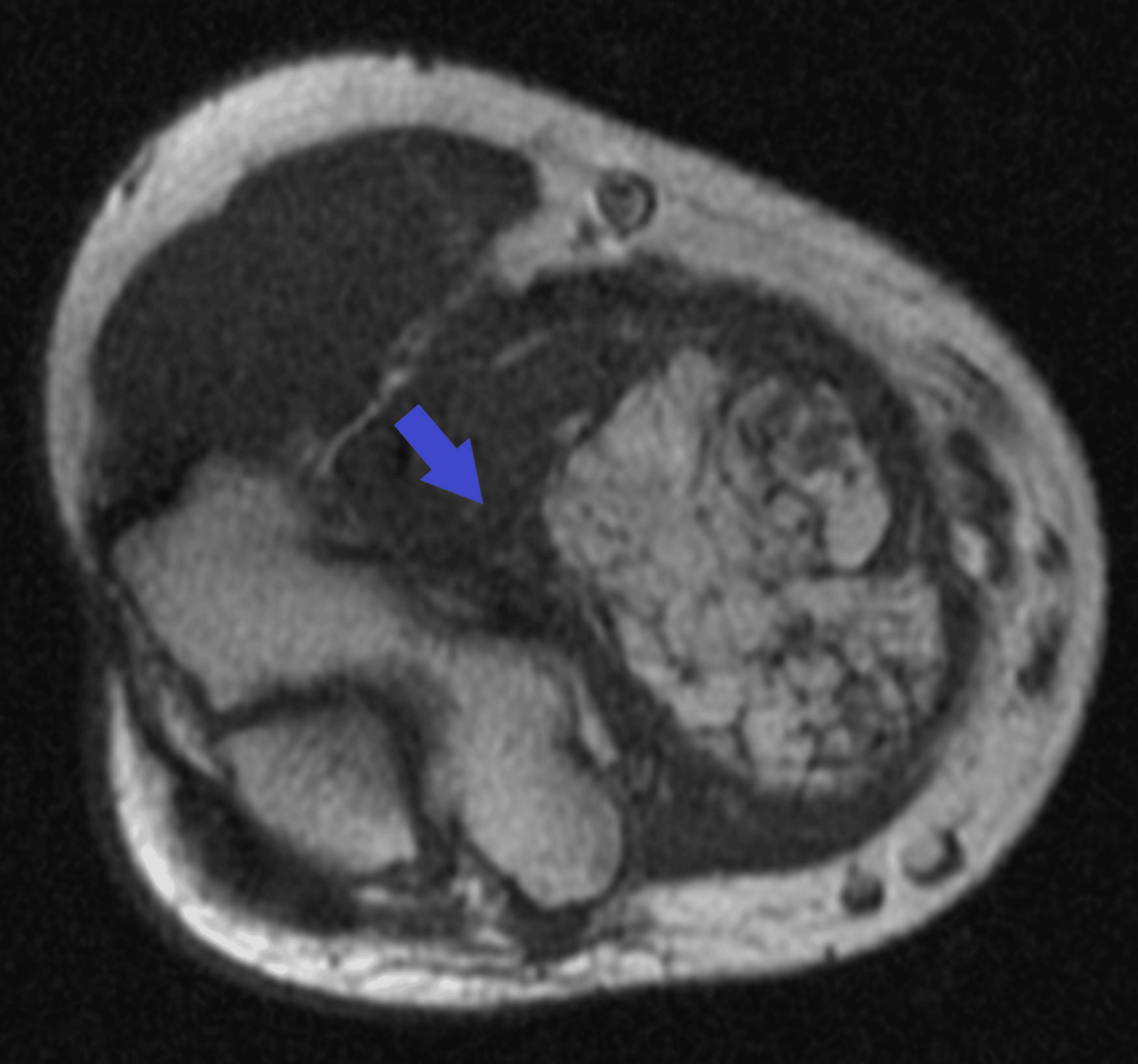
T2 weighted MRI image of right upper extremity showing a heterogeneous soft tissue with bone abutment (blue arrow)

A 30-year-old male patient, who initially reported a small mass in his right brachial region five years earlier, presented with a notable acceleration in growth within the last year, prompting the need for medical intervention. Physical examination identified a large, soft, non-mobile, non-tender 10 cm mass at the medial aspect of the right upper extremity (RUE). There was full range of motion with no overlying skin changes. Magnetic resonance imaging (MRI) of the RUE demonstrated a medial compartment heterogeneous mass with abnormal fluid intake and mixed fatty/solid components along the right brachialis muscle, measuring 12 x 7 x 6 cm, concerning for a liposarcoma (Figure 1) and prompting the need for a biopsy.

Preliminary pathology revealed a moderately cellular specimen consisting of atypical spindle and epithelioid cells with hyperchromasia and nuclear pleomorphism with variable collagenous stroma. Ki-67 labeling was focally increased. The tumor was diffusely strongly positive for STAT6 (signal transducer and activator of transcription 6), focally positive for desmin, and negative for cytokeratin AE1/3, CD34, ERG (ETS-related gene), beta-catenin, S100, and smooth muscle actin (SMA). Next-generation sequencing study revealed amplification of the adjacent genes STAT6, CDK4 (cyclin-dependent kinase 4), and MDM2 (murine double minute 2) but no evidence of fusion involving STAT6. Final pathology analysis of the biopsy with immunochemistry was positive for MDM2. There was also a positive amplification of MDM2 with RNA in situ hybridization. The pathology findings led to a diagnosis of DDL of intermediate grade with Fédération Nationale des Centres de Lutte Contre le Cancer (FNCLCC) grade 2-3.

**Figure 2:**
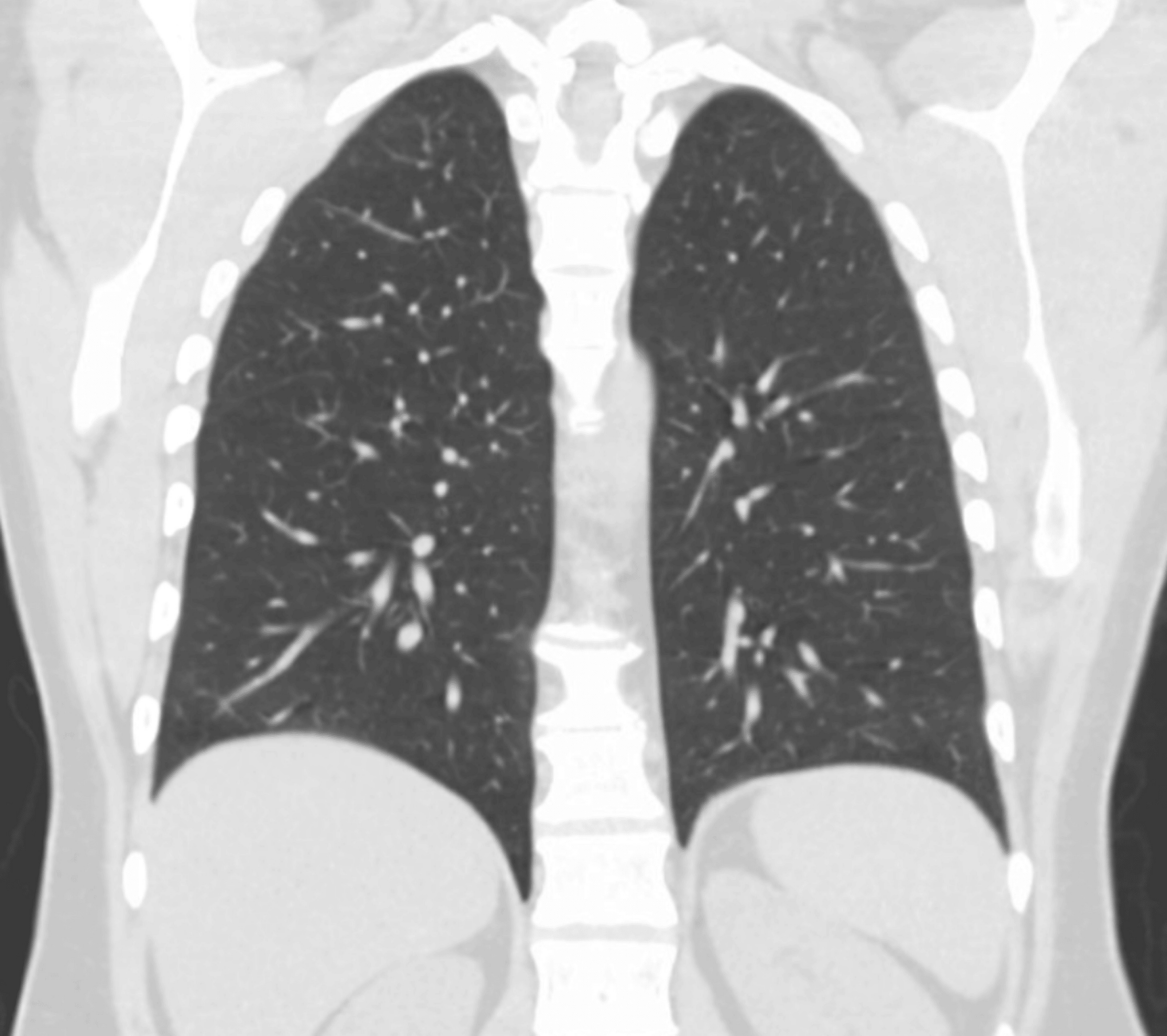
Coronal view of CT chest showing no signs of metastatic disease.

The decision was made to proceed with surgery, with the potential inclusion of adjuvant chemotherapy. The preoperative staging was completed with computed tomography (CT) of the chest showing no evidence of thoracic metastatic disease as shown in Figure 2.

**Figure 3:**
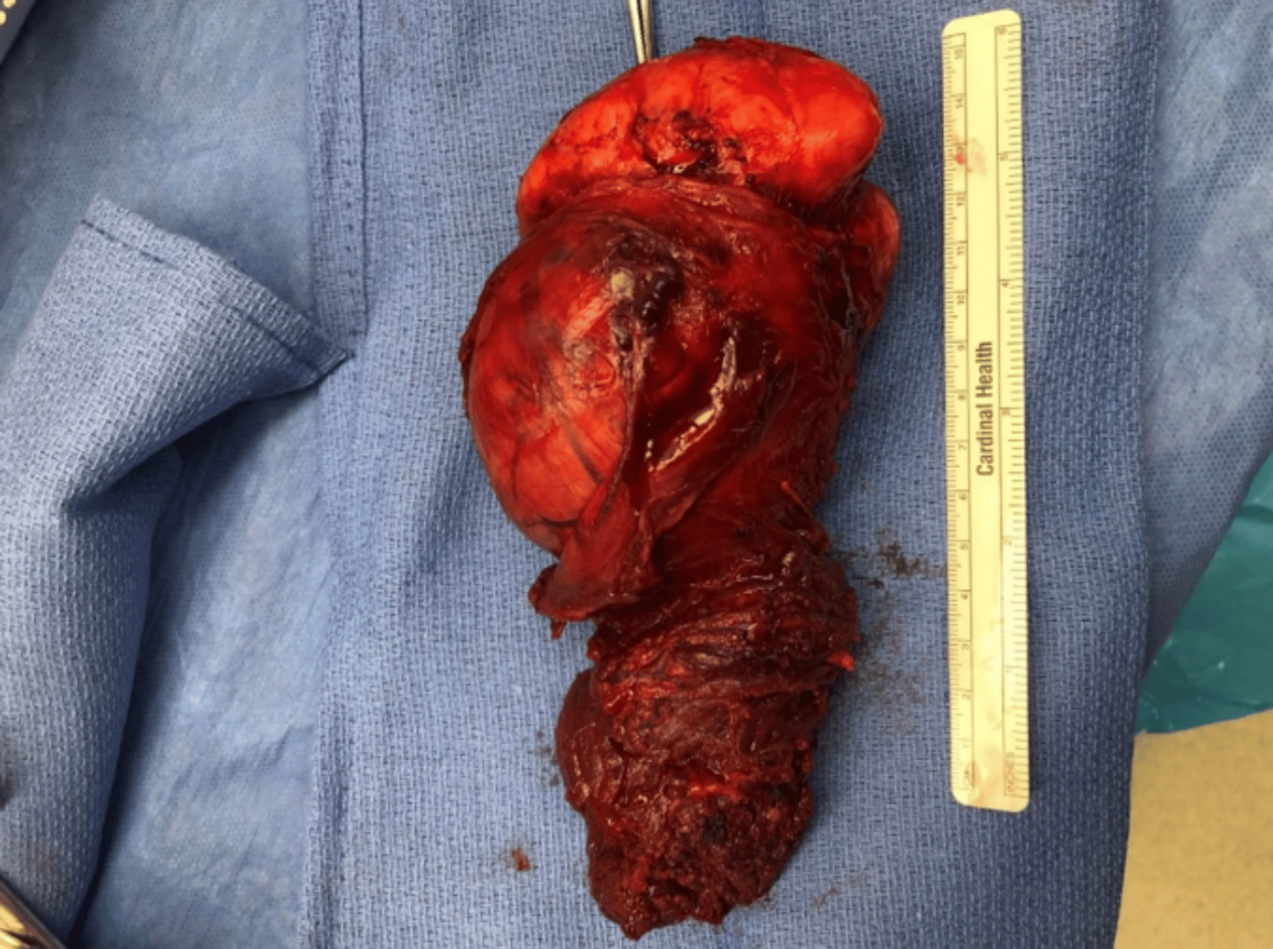
Gross image of the dedifferentiated liposarcoma.

**Figure 4:**
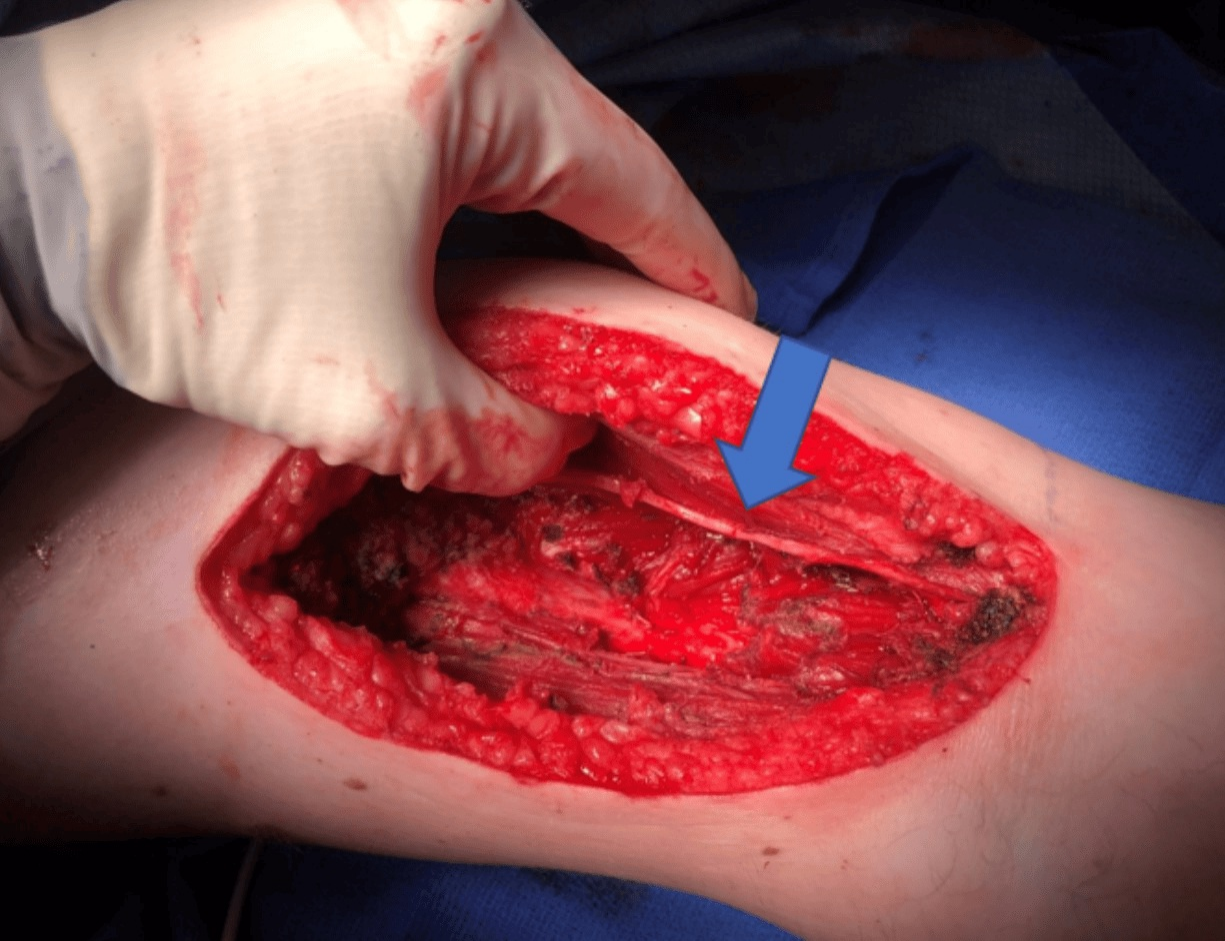
Wound bed status post tumor resection with the median nerve visible (blue arrow).

Operatively, a right anterior compartment radical resection of the brachialis muscle was performed. Once there was full exposure of the origin site of the muscle at the anterior aspect of the humerus near the deltoid, the brachialis muscle was divided away from the mass. A cartilaginous structure in the antecubital fossa was identified as part of the mass, which was then excised en bloc with the brachialis muscle, and distal division occurred at the ulnar tuberosity insertion site. The mass (Figure 3), marked at the ulnar aspect, was sent for pathology. The patient was neurovascularly intact at the end of the procedure moving his arm hand with adequate flexion and palpable brachial and radial pulses. The wound bed after tumor resection is shown in Figure 4.

Pathological analysis revealed a mass of skeletal muscle measuring 12.0 x 6.5 x 5.0 cm. Protruding from the medial aspect of the muscle was an 11.0 x 8.0 x 6.5 cm thinly encapsulated, solid, ovoid, slightly lobulated, rubbery to hard, tan mass. The mass appeared to arise from the muscular parenchyma. The bony component comprised approximately 25% of the overall mass volume. There were no areas of degeneration, hemorrhage, and/or infarction. The diagnosis was confirmed as DDL with intermediate grade (FNCLCC grade 2) extending to a distal surgical margin.

The patient was discharged home on postoperative day 1 in stable condition with instructions for an outpatient follow-up. Follow-up care with a PET scan showed uptake in the area of the resection. There was no evidence of regional axillary adenopathy or other metastatic disease in the chest, abdomen, and pelvis. The patient was subsequently prescribed adjuvant chemoradiation therapy with doxorubicin and pazopanib.

## Discussion

Liposarcomas exhibit diverse histological patterns and morphological presentations. DDL represents a manifestation of disease progression and is characterized by the juxtaposition of well-differentiated liposarcoma with a high-grade, pleomorphic sarcoma [6]. Dedifferentiation may occur either as a de novo occurrence (primary dedifferentiation) or later in the course of a prior well-differentiated liposarcoma (secondary dedifferentiation). DDL usually arises in the retroperitoneum and is uncommon in the extremities. DDL is clinically less aggressive than other high-grade sarcomas with the reported rate of local recurrence, distant metastasis, and disease-related mortality of 41%, 17%, and 28%, respectively [6].

Tumor anatomic location is the most important prognostic factor with retroperitoneal tumors having the worst outcomes due to challenges with complete excision [6]. Rapid changes in a long-standing tumor are a clinical sign of dedifferentiation [3,7]. The predominant form of dedifferentiation in DDL often manifests as a high-grade tumor, typically resembling malignant fibrous histiocytoma (MFH) or fibrosarcoma [6]. However, dedifferentiated areas can encompass a variety of transformations resembling the patterns seen in carcinoma, melanoma, or anaplastic lymphoma. Approximately 10% of DDL cases exhibit dedifferentiation that diverges from the MFH or fibrosarcoma patterns to areas with rhabdomyosarcomatous, leiomyosarcomatous, or osteosarcomatous features [6]. Cases of metaplastic bone formation are relatively rare [8].

Similar to the patient in the present report, in an analysis of nine DDL cases by Nascimento et al., all tumors with an osseous component showed that the bone was intimately associated with the cellular element [9]. The bone in these cases was considered to be likely benign and hypothesized to arise as a result of metaplastic change. The genetic hallmark of DDL is amplification of the 12q13-15 region which contains MDM2 and CDK4 [10]. The standard procedure to identify a sarcoma includes the detection of this amplification through fluorescence in situ hybridization (FISH) and/or MDM2/CDK4 immunohistochemistry [2]. Analysis of this patient’s tumor showed high levels of amplification of MDM2 and CDK4 likely indicating that despite the unique morphological features, bone formation shared many of the cytogenetic features of the common forms of DDL.

The standard treatment for DDL is wide resection with microscopically margin-negative resection (R0 margin) [11]. Determination of the most suitable procedure for a patient relies on various factors including tumor location, size, stage, proximity to surrounding neurovascular and bony elements, as well as considerations for both functional and cosmetic outcomes. Patients with DDL in the extremities may benefit from adjuvant radiation therapy when complete resection is limited by proximity to major nerves and vessels [11].

## Conclusions

DDL has a high metastatic potential irrespective of its location. It is therefore crucial to distinguish benign bone formation from osteosarcomatous differentiation in DDL. More research is needed to understand the molecular events associated with ossification in DDL. Complete resection of DDLs should always be performed to reduce the potential of metastasis. Diligent monitoring during the follow-up period is crucial, especially in instances of DDL affecting the extremities and patients should be evaluated for adjuvant therapy.
